# (*μ*-Formato-*κ* ^2^ *O*:*O*′)bis­[dicarbon­yl(*η* ^5^-cyclo­penta­dien­yl)iron(II)] tetra­fluoridoborate

**DOI:** 10.1107/S1600536811032764

**Published:** 2011-08-17

**Authors:** Cyprian M. M’thiruaine, Holger B. Friedrich, Evans O. Changamu, Bernard Omondi

**Affiliations:** a School of Chemistry, University of KwaZulu-Natal, Westville Campus, Private Bag X54001, Durban 4000, South Africa; b Chemistry Department, Kenyatta University, PO Box 43844, Nairobi, Kenya

## Abstract

In the structure of the title compound [Fe_2_(C_5_H_5_)_2_(CHO_2_)(CO)_4_]BF_4_, each Fe^II^ atom is coordinated in a pseudo-octa­hedral three-legged piano-stool fashion. The cyclo­penta­dienyl ligand occupies three *fac* coordination sites while the two carbonyl ligands and formate O atom occupy the remaining three sites.

## Related literature

For the synthesis of the title and other analogous compounds, see: Tso & Cutler (1985 ▶, 1990 ▶). For mononuclear [Fe(*κ* ^1^-OCHO)(*η* ^5^-C_5_H_5_)(CO)_2_], see: Darensbourg, Day *et al.* (1981 ▶); Darensbourg, Fischer *et al.* (1981 ▶); Dombek & Angelici (1973 ▶). For related compounds, see: M’thiruaine, Friedrich, Changamu & Bala (2011 ▶); M’thiruaine, Friedrich, Changamu & Omondi (2011 ▶)*;* Pinkes *et al.* (1997 ▶).
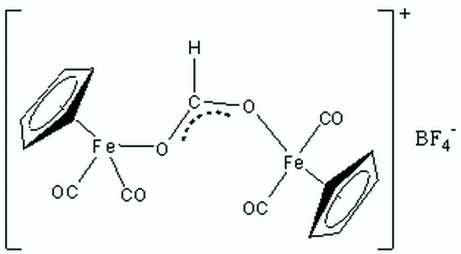


## Experimental

### 

#### Crystal data

[Fe_2_(C_5_H_5_)_2_(CHO_2_)(CO)_4_]BF_4_
*M* *_r_* = 485.75
Monoclinic, 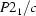

*a* = 7.4964 (5) Å
*b* = 17.8845 (14) Å
*c* = 14.1931 (9) Å
β = 115.144 (3)°
*V* = 1722.5 (2) Å^3^
*Z* = 4
Mo *K*α radiation
μ = 1.76 mm^−1^
*T* = 100 K
0.24 × 0.11 × 0.1 mm

#### Data collection

Bruker X8 APEXII 4K Kappa CCD diffractometer
Absorption correction: multi-scan (*SADABS*; Bruker, 2007 ▶) *T*_min_ = 0.678, *T*_max_ = 0.844
41366 measured reflections
4341 independent reflections
3784 reflections with *I* > 2σ(*I*)
*R*_int_ = 0.048

#### Refinement

*R*[*F* ^2^ > 2σ(*F* ^2^)] = 0.028
*wR*(*F* ^2^) = 0.069
*S* = 1.02
4341 reflections
253 parameters
11 restraints
H-atom parameters constrained
Δρ_max_ = 1.38 e Å^−3^
Δρ_min_ = −0.92 e Å^−3^

### 

Data collection: *APEX2* (Bruker, 2007 ▶); cell refinement: *SAINT-Plus* (Bruker, 2007 ▶); data reduction: *SAINT-Plus* and *XPREP* (Bruker, 2007 ▶); program(s) used to solve structure: *SHELXS97* (Sheldrick, 2008 ▶); program(s) used to refine structure: *SHELXL97* (Sheldrick, 2008 ▶); molecular graphics: *ORTEP-3* (Farrugia, 1997 ▶); software used to prepare material for publication: *WinGX* (Farrugia, 1999 ▶).

## Supplementary Material

Crystal structure: contains datablock(s) global, I. DOI: 10.1107/S1600536811032764/ng5211sup1.cif

Structure factors: contains datablock(s) I. DOI: 10.1107/S1600536811032764/ng5211Isup2.hkl

Additional supplementary materials:  crystallographic information; 3D view; checkCIF report

## Figures and Tables

**Fig. 1.**
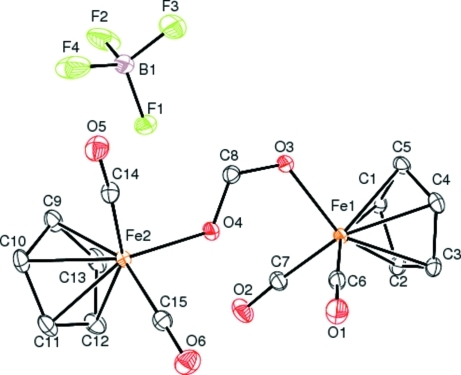
View of (I) (50% probability displacement ellipsoids) with H atoms omited for clarity.

## supplementary crystallographic information

### Comment

There has been a considerable interest in metalloformates and metallocarboxylates due to their potential application in the catalysis of water-gas shift reactions (Darensbourg, Day *et al.* 1981; Darensbourg, Fischer *et al.* 1981) and catalytic reduction of CO_2_ (Tso & Cutler, 1985, 1990; Pinkes *et al.* 1997). In connection to this the neutral mononuclear formate complex [(*η*^5^-C_5_H_5_)Fe(CO)_2_(*η*^1^-OC(H)O)] has been prepared using different routes (Dombek & Angelici 1973; Darensbourg, Day *et al.* 1981; Tso & Cutler, 1985) and its molecular structure is well known (Darensbourg, Day *et al.* 1981; Darensbourg, Fischer *et al.* 1981). The cationic binuclear complex [{(*η*^5^-C_5_H_5_)Fe(CO)_2_}_2_(*µ*-OC(H)O)]PF_6_ has been reported as the product of the reaction between the neutral mononuclear complex [(*η*^5^-C_5_H_5_)Fe(CO)_2_(*η*^1^-OC(H)O)] and [(*η*_5_-C_5_H_5_)Fe(CO)_2_(THF)]PF_6_, and has been assumed to exist as a *syn*-*syn* isomer based on spectroscopic data (Tso & Cutler, 1985). The same authors have reported various formate bridged heterobimetallic complexes (Tso & Cutler, 1990) but none of their crystal structures are known.

The title compound (I) was obtained in high yields from the reaction of formic acid with two equivalents of the diethyl ether complex [(*η*^5^-C_5_H_5_)Fe(CO)_2_(O(CH_2_CH_3_)_2_)]BF_4_. This is a part of our study on the reactions of the diethyl ether complex with electron pair donor ligands (M'thiruaine, Friedrich, Changamu & Bala, 2011; M'thiruaine, Friedrich, Changamu & Omondi, 2011). The crystallizes with one discrete molecular cation and one counter anion in the assymetric unit. Each Fe atom is coordinated in a pseudo-octahedral three-legged piano stool fashion in which the iron metal capped with cyclopentadienyl occupies three coordination sites while the two carbonyl ligands and formate oxygen occupy the other three coordination sites (Fig. 1). The Fe—O bond lengths of 1.9844 (13) and 1.9686 (13) Å are close to the 1.957 (2)Å reported for the neutral mononuclear complex [(*η*^5^-C_5_H_5_)Fe(CO)_2_(*η*^1^-OC(H)O)] (Darensbourg, Day *et al.* 1981). The two O—C bonds of the formate group (–OC(H)O–) are identical, with the bond distances being equal to 1.256 (2) and 1.258 (2) Å, which is close to 1.277 (3)Å and 1.208 (4) found for coordinated and uncoordinated O—C of the formate moiety in the complex [(*η*^5^-C_5_H_5_)Fe(CO)_2_(*η*^1^-OC(H)O)], respectively. The identical bond lengths of the two C—O bonds of bridging formate indicate electron delocalization between the two oxygen atoms of the formate moiety. Thus the structure shown in Fig. 1 is an overall structure of two resonance structures: [Fp—O=C(H)O—Fp]^+^ and [Fp—O-(H)C=O—Fp]^+^. This greatly contributes to the stability of the title compound in both solution and solid state. The Fp moieties are oriented in the solid state so as to adopt a *syn*-*anti* isomer structure contrary to the assumption made by Tso & Cutler (1985).

### Experimental

The compound was synthesized as described below and its spectroscopic data is in good agreement with data reported for the PF_6_^-^ salt.

To a solution of [(*η*^5^-C_5_H_5_)Fe(CO)_2_(O(CH_2_CH_3_)_2_)]BF_4_ (0.560 g, 1.66 mmol) in CH_2_Cl_2_ (10 ml), 98% formic acid (0.030 ml, 0.796 mmol) was added and the mixture stirred at room temperature for 5 h after which diethyl ether was added to precipitate the formate compound as a light red solid. The mixture was allowed to stand for 30 min and then the mother liquor was syringed off and the residue washed with (2 *x* 5 ml) diethyl to give 0.70 g (87% yield) of the light red solid. Anal. Calc. for C_15_H_11_BF_4_Fe_2_O_6_: C, 37.09; H, 2.28% Found: C, 36.53; H, 2.57%. ^1^H NMR (400 MHz, acetone-*d*_6_): δ 5.46 (s, 10H, Cp), 7.18 (s, 1H, OCHO). ^13^C NMR (400 MHz, acetone-*d*_6_): δ 86.88 (Cp) 212.23 (CO). IR (solid state): ν(CO) 2057, 2039, 1985 cm^-1^, v(OCO) 1562 cm^-1^. *M*.p 109–110 °C.

### Refinement

All H atoms were placed in geometrically idealized positions and constrained to ride on their parent atoms, with C—H = 0.95–1.00 Å and with *U*_iso_(H) = 1.2*U*_eq_(C).

### Crystal data

**Table:**

[Fe_2_(C_5_H_5_)_2_(CHO_2_)(CO)_4_]BF_4_	*F*(000) = 968
*M**_r_* = 485.75	*D*_x_ = 1.873 Mg m^−^^3^
Monoclinic, *P*2_1_/*c*	Mo *K*α radiation, λ = 0.71073 Å
Hall symbol: -P 2ybc	Cell parameters from 42124 reflections
*a* = 7.4964 (5) Å	θ = 2.0–28.5°
*b* = 17.8845 (14) Å	µ = 1.76 mm^−^^1^
*c* = 14.1931 (9) Å	*T* = 100 K
β = 115.144 (3)°	Block, brown
*V* = 1722.5 (2) Å^3^	0.24 × 0.11 × 0.1 mm
*Z* = 4	

### Data collection

**Table:**

Bruker X8 APEXII 4K Kappa CCD diffractometer	3784 reflections with *I* > 2σ(*I*)
graphite	*R*_int_ = 0.048
φ and ω scans	θ_max_ = 28.5°, θ_min_ = 2.0°
Absorption correction: multi-scan (*SADABS*; Bruker, 2007)	*h* = −9→10
*T*_min_ = 0.678, *T*_max_ = 0.844	*k* = −24→23
41366 measured reflections	*l* = −19→19
4341 independent reflections	

### Refinement

**Table:**

Refinement on *F*^2^	Primary atom site location: structure-invariant direct methods
Least-squares matrix: full	Secondary atom site location: difference Fourier map
*R*[*F*^2^ > 2σ(*F*^2^)] = 0.028	Hydrogen site location: inferred from neighbouring sites
*wR*(*F*^2^) = 0.069	H-atom parameters constrained
*S* = 1.02	*w* = 1/[σ^2^(*F*_o_^2^) + (0.0257*P*)^2^ + 2.6185*P*] where *P* = (*F*_o_^2^ + 2*F*_c_^2^)/3
4341 reflections	(Δ/σ)_max_ = 0.002
253 parameters	Δρ_max_ = 1.38 e Å^−^^3^
11 restraints	Δρ_min_ = −0.92 e Å^−^^3^

### Special details

**Table:**

Experimental. All H atoms were placed in geometrically idealized positions and constrained to ride on their parent atoms, with C—H = 0.95–1.00 Å and with *U*_iso_(H) = 1.2*U*_eq_(C).
Geometry. All e.s.d.'s (except the e.s.d. in the dihedral angle between two l.s. planes) are estimated using the full covariance matrix. The cell e.s.d.'s are taken into account individually in the estimation of e.s.d.'s in distances, angles and torsion angles; correlations between e.s.d.'s in cell parameters are only used when they are defined by crystal symmetry. An approximate (isotropic) treatment of cell e.s.d.'s is used for estimating e.s.d.'s involving l.s. planes.
Refinement. Refinement of *F*^2^ against ALL reflections. The weighted *R*-factor *wR* and goodness of fit *S* are based on *F*^2^, conventional *R*-factors *R* are based on *F*, with *F* set to zero for negative *F*^2^. The threshold expression of *F*^2^ > σ(*F*^2^) is used only for calculating *R*-factors(gt) *etc*. and is not relevant to the choice of reflections for refinement. *R*-factors based on *F*^2^ are statistically about twice as large as those based on *F*, and *R*- factors based on ALL data will be even larger. 244_ALERT_4_C Low 'Solvent' *U*_eq_ as Compared to Neighbors for B1 912_ALERT_4_C Missing # of FCF Reflections Above STh/*L*= 0.600 26 003_ALERT_ _G # Space-Group NOTED. 232_ALERT_2_G Hirshfeld Test Diff (M—*X*) Fe1 – C7.. 5.63 su 232_ALERT_2_G Hirshfeld Test Diff (M—*X*) Fe2 – C14.. 5.38 su 232_ALERT_2_G Hirshfeld Test Diff (M—*X*) Fe2 – C15.. 5.63 su DELU and SIMU restraints used. 790_ALERT_4_G Centre of Gravity not Within Unit Cell: Resd. # 2 B F4 860_ALERT_3_G Note: Number of Least-Squares Restraints ······. 11 NOTED.

### Fractional atomic coordinates and isotropic or equivalent isotropic displacement parameters (Å^2^)

**Table:**

	*x*	*y*	*z*	*U*_iso_*/*U*_eq_	
C1	−0.3243 (3)	0.92926 (11)	0.07899 (14)	0.0147 (4)	
H1	−0.3337	0.88	0.0438	0.018*	
C2	−0.1969 (3)	0.99018 (11)	0.08289 (14)	0.0150 (4)	
H2	−0.1025	0.9911	0.05	0.018*	
C3	−0.2325 (3)	1.04957 (11)	0.13869 (14)	0.0153 (4)	
H3	−0.1677	1.0998	0.1522	0.018*	
C4	−0.3832 (3)	1.02592 (11)	0.16890 (14)	0.0152 (4)	
H4	−0.44	1.0562	0.2088	0.018*	
C5	−0.4380 (3)	0.95243 (11)	0.13173 (14)	0.0153 (4)	
H5	−0.5392	0.9214	0.1421	0.018*	
C6	0.0115 (3)	1.01353 (10)	0.34237 (14)	0.0146 (3)	
C7	0.0791 (3)	0.90816 (11)	0.23422 (15)	0.0161 (3)	
C8	−0.0601 (3)	0.85414 (10)	0.39906 (14)	0.0137 (3)	
H8	−0.0936	0.8167	0.4362	0.016*	
C9	0.3687 (3)	0.72050 (11)	0.53224 (15)	0.0179 (4)	
H9	0.2698	0.6817	0.5283	0.021*	
C10	0.5315 (3)	0.74607 (11)	0.62519 (15)	0.0175 (4)	
H10	0.5666	0.7279	0.6975	0.021*	
C11	0.6362 (3)	0.79995 (12)	0.59592 (16)	0.0188 (4)	
H11	0.7579	0.8268	0.644	0.023*	
C12	0.5394 (3)	0.80858 (12)	0.48493 (16)	0.0189 (4)	
H12	0.5807	0.8426	0.4419	0.023*	
C13	0.3781 (3)	0.75852 (11)	0.44746 (15)	0.0185 (4)	
H13	0.2831	0.7523	0.3728	0.022*	
C14	0.2328 (3)	0.82195 (10)	0.63946 (15)	0.0151 (3)	
C15	0.4344 (3)	0.92972 (11)	0.59175 (14)	0.0150 (3)	
B1	−0.1687 (3)	0.64951 (14)	0.37678 (18)	0.0201 (4)	
F1	−0.06516 (18)	0.69395 (7)	0.33590 (9)	0.0219 (3)	
F2	−0.2175 (2)	0.58267 (8)	0.32135 (15)	0.0431 (4)	
F3	−0.3406 (2)	0.68494 (8)	0.36527 (13)	0.0340 (3)	
F4	−0.0473 (2)	0.63435 (12)	0.47950 (12)	0.0510 (5)	
Fe1	−0.13319 (4)	0.955840 (14)	0.233347 (19)	0.01069 (7)	
Fe2	0.34774 (4)	0.835389 (14)	0.552967 (19)	0.01083 (7)	
O1	0.1019 (2)	1.05243 (8)	0.40890 (12)	0.0232 (3)	
O2	0.2129 (2)	0.88085 (9)	0.23040 (12)	0.0250 (3)	
O3	−0.19470 (19)	0.88025 (8)	0.31758 (10)	0.0149 (3)	
O4	0.11643 (19)	0.87505 (7)	0.43390 (10)	0.0139 (3)	
O5	0.1671 (2)	0.80908 (9)	0.69662 (11)	0.0228 (3)	
O6	0.4921 (2)	0.98874 (8)	0.61515 (12)	0.0241 (3)	

### Atomic displacement parameters (Å^2^)

**Table:**

	*U*^11^	*U*^22^	*U*^33^	*U*^12^	*U*^13^	*U*^23^
C1	0.0144 (9)	0.0155 (9)	0.0115 (8)	0.0002 (7)	0.0029 (7)	−0.0001 (7)
C2	0.0149 (9)	0.0174 (9)	0.0111 (8)	0.0007 (7)	0.0042 (7)	0.0031 (7)
C3	0.0153 (9)	0.0138 (8)	0.0145 (8)	0.0018 (7)	0.0041 (7)	0.0036 (7)
C4	0.0123 (8)	0.0191 (9)	0.0121 (8)	0.0046 (7)	0.0031 (7)	0.0021 (7)
C5	0.0101 (8)	0.0201 (9)	0.0132 (8)	0.0011 (7)	0.0023 (7)	0.0035 (7)
C6	0.0135 (9)	0.0144 (8)	0.0140 (8)	0.0020 (6)	0.0038 (7)	0.0019 (6)
C7	0.0138 (8)	0.0185 (9)	0.0153 (8)	0.0018 (6)	0.0055 (7)	0.0015 (7)
C8	0.0148 (9)	0.0133 (8)	0.0142 (8)	0.0011 (7)	0.0074 (7)	0.0012 (7)
C9	0.0151 (9)	0.0130 (9)	0.0222 (9)	0.0028 (7)	0.0046 (8)	−0.0033 (7)
C10	0.0152 (9)	0.0150 (9)	0.0188 (9)	0.0057 (7)	0.0038 (7)	0.0005 (7)
C11	0.0111 (9)	0.0205 (9)	0.0218 (9)	0.0037 (7)	0.0041 (7)	−0.0013 (8)
C12	0.0155 (9)	0.0238 (10)	0.0208 (9)	0.0031 (8)	0.0109 (8)	−0.0021 (8)
C13	0.0167 (9)	0.0212 (10)	0.0161 (9)	0.0061 (8)	0.0053 (7)	−0.0053 (7)
C14	0.0159 (9)	0.0145 (8)	0.0147 (8)	0.0029 (7)	0.0063 (7)	0.0013 (7)
C15	0.0143 (9)	0.0170 (7)	0.0130 (8)	0.0005 (7)	0.0051 (7)	−0.0005 (6)
B1	0.0165 (11)	0.0222 (11)	0.0202 (10)	−0.0014 (9)	0.0064 (9)	0.0029 (9)
F1	0.0215 (6)	0.0243 (6)	0.0217 (6)	−0.0017 (5)	0.0110 (5)	0.0033 (5)
F2	0.0262 (8)	0.0265 (7)	0.0816 (12)	−0.0079 (6)	0.0275 (8)	−0.0190 (8)
F3	0.0321 (8)	0.0294 (7)	0.0534 (9)	0.0073 (6)	0.0307 (7)	0.0015 (6)
F4	0.0333 (9)	0.0855 (13)	0.0248 (7)	−0.0234 (9)	0.0032 (6)	0.0205 (8)
Fe1	0.00883 (12)	0.01172 (13)	0.01071 (12)	0.00072 (9)	0.00336 (10)	0.00103 (9)
Fe2	0.00995 (13)	0.01173 (13)	0.01013 (12)	0.00130 (9)	0.00361 (10)	0.00004 (9)
O1	0.0228 (8)	0.0187 (7)	0.0208 (7)	−0.0012 (6)	0.0020 (6)	−0.0026 (6)
O2	0.0186 (7)	0.0328 (9)	0.0259 (8)	0.0080 (6)	0.0118 (6)	0.0032 (6)
O3	0.0112 (6)	0.0164 (6)	0.0152 (6)	0.0000 (5)	0.0037 (5)	0.0038 (5)
O4	0.0118 (6)	0.0156 (6)	0.0129 (6)	0.0012 (5)	0.0039 (5)	0.0022 (5)
O5	0.0274 (8)	0.0251 (8)	0.0212 (7)	0.0040 (6)	0.0153 (7)	0.0045 (6)
O6	0.0277 (8)	0.0187 (7)	0.0245 (7)	−0.0037 (6)	0.0097 (7)	−0.0031 (6)

### Geometric parameters (Å, °)

**Table:**

C1—C5	1.414 (3)	C9—Fe2	2.0910 (19)
C1—C2	1.434 (3)	C9—H9	1
C1—Fe1	2.1002 (18)	C10—C11	1.412 (3)
C1—H1	1	C10—Fe2	2.0737 (19)
C2—C3	1.416 (3)	C10—H10	0.9999
C2—Fe1	2.0732 (18)	C11—C12	1.435 (3)
C2—H2	1	C11—Fe2	2.0805 (19)
C3—C4	1.432 (3)	C11—H11	0.9999
C3—Fe1	2.0783 (19)	C12—C13	1.414 (3)
C3—H3	1	C12—Fe2	2.0990 (19)
C4—C5	1.411 (3)	C12—H12	1.0001
C4—Fe1	2.1125 (19)	C13—Fe2	2.1152 (19)
C4—H4	1.0001	C13—H13	1.0001
C5—Fe1	2.1224 (19)	C14—O5	1.137 (2)
C5—H5	1	C14—Fe2	1.7906 (19)
C6—O1	1.136 (2)	C15—O6	1.135 (2)
C6—Fe1	1.7915 (19)	C15—Fe2	1.808 (2)
C7—O2	1.138 (2)	B1—F4	1.378 (3)
C7—Fe1	1.8009 (19)	B1—F3	1.382 (3)
C8—O4	1.256 (2)	B1—F2	1.392 (3)
C8—O3	1.258 (2)	B1—F1	1.397 (3)
C8—H8	0.95	Fe1—O3	1.9844 (13)
C9—C13	1.410 (3)	Fe2—O4	1.9686 (13)
C9—C10	1.439 (3)		
			
C5—C1—C2	107.34 (17)	O6—C15—Fe2	178.34 (18)
C5—C1—Fe1	71.28 (11)	F4—B1—F3	112.2 (2)
C2—C1—Fe1	68.89 (10)	F4—B1—F2	109.0 (2)
C5—C1—H1	126.3	F3—B1—F2	108.60 (18)
C2—C1—H1	126.3	F4—B1—F1	108.53 (18)
Fe1—C1—H1	126.3	F3—B1—F1	110.15 (18)
C3—C2—C1	108.10 (16)	F2—B1—F1	108.22 (18)
C3—C2—Fe1	70.24 (10)	C6—Fe1—C7	93.30 (9)
C1—C2—Fe1	70.92 (10)	C6—Fe1—O3	94.73 (7)
C3—C2—H2	125.9	C7—Fe1—O3	95.98 (7)
C1—C2—H2	125.9	C6—Fe1—C2	120.60 (8)
Fe1—C2—H2	125.9	C7—Fe1—C2	87.91 (8)
C2—C3—C4	107.81 (17)	O3—Fe1—C2	144.23 (7)
C2—C3—Fe1	69.86 (10)	C6—Fe1—C3	90.74 (8)
C4—C3—Fe1	71.32 (11)	C7—Fe1—C3	117.34 (8)
C2—C3—H3	126.1	O3—Fe1—C3	145.87 (7)
C4—C3—H3	126.1	C2—Fe1—C3	39.90 (7)
Fe1—C3—H3	126.1	C6—Fe1—C1	157.78 (8)
C5—C4—C3	107.74 (17)	C7—Fe1—C1	96.27 (8)
C5—C4—Fe1	70.92 (11)	O3—Fe1—C1	104.12 (7)
C3—C4—Fe1	68.74 (11)	C2—Fe1—C1	40.19 (7)
C5—C4—H4	126.1	C3—Fe1—C1	67.05 (7)
C3—C4—H4	126.1	C6—Fe1—C4	97.62 (8)
Fe1—C4—H4	126.1	C7—Fe1—C4	154.51 (8)
C4—C5—C1	109.00 (17)	O3—Fe1—C4	105.94 (7)
C4—C5—Fe1	70.16 (11)	C2—Fe1—C4	66.70 (7)
C1—C5—Fe1	69.59 (11)	C3—Fe1—C4	39.94 (7)
C4—C5—H5	125.5	C1—Fe1—C4	66.18 (7)
C1—C5—H5	125.5	C6—Fe1—C5	133.28 (8)
Fe1—C5—H5	125.5	C7—Fe1—C5	133.17 (8)
O1—C6—Fe1	177.30 (17)	O3—Fe1—C5	86.05 (7)
O2—C7—Fe1	176.01 (18)	C2—Fe1—C5	66.31 (7)
O4—C8—O3	123.42 (17)	C3—Fe1—C5	66.25 (8)
O4—C8—H8	118.3	C1—Fe1—C5	39.13 (7)
O3—C8—H8	118.3	C4—Fe1—C5	38.92 (7)
C13—C9—C10	107.38 (18)	C14—Fe2—C15	97.58 (9)
C13—C9—Fe2	71.35 (11)	C14—Fe2—O4	97.58 (7)
C10—C9—Fe2	69.14 (11)	C15—Fe2—O4	89.81 (7)
C13—C9—H9	126.3	C14—Fe2—C10	88.27 (8)
C10—C9—H9	126.3	C15—Fe2—C10	119.41 (8)
Fe2—C9—H9	126.3	O4—Fe2—C10	149.28 (7)
C11—C10—C9	107.94 (18)	C14—Fe2—C11	120.08 (9)
C11—C10—Fe2	70.39 (11)	C15—Fe2—C11	90.05 (9)
C9—C10—Fe2	70.43 (11)	O4—Fe2—C11	142.00 (7)
C11—C10—H10	126	C10—Fe2—C11	39.74 (8)
C9—C10—H10	126	C14—Fe2—C9	92.94 (8)
Fe2—C10—H10	126	C15—Fe2—C9	157.11 (8)
C10—C11—C12	108.14 (18)	O4—Fe2—C9	108.95 (7)
C10—C11—Fe2	69.87 (11)	C10—Fe2—C9	40.43 (8)
C12—C11—Fe2	70.61 (11)	C11—Fe2—C9	67.11 (8)
C10—C11—H11	125.9	C14—Fe2—C12	155.18 (8)
C12—C11—H11	125.9	C15—Fe2—C12	97.22 (8)
Fe2—C11—H11	125.9	O4—Fe2—C12	102.29 (7)
C13—C12—C11	107.30 (18)	C10—Fe2—C12	67.08 (8)
C13—C12—Fe2	71.01 (11)	C11—Fe2—C12	40.17 (8)
C11—C12—Fe2	69.22 (11)	C9—Fe2—C12	66.65 (8)
C13—C12—H12	126.4	C14—Fe2—C13	128.76 (9)
C11—C12—H12	126.3	C15—Fe2—C13	133.58 (8)
Fe2—C12—H12	126.3	O4—Fe2—C13	86.96 (7)
C9—C13—C12	109.22 (17)	C10—Fe2—C13	66.45 (8)
C9—C13—Fe2	69.49 (11)	C11—Fe2—C13	66.32 (8)
C12—C13—Fe2	69.77 (11)	C9—Fe2—C13	39.16 (8)
C9—C13—H13	125.4	C12—Fe2—C13	39.21 (8)
C12—C13—H13	125.4	C8—O3—Fe1	120.38 (12)
Fe2—C13—H13	125.4	C8—O4—Fe2	128.81 (12)
O5—C14—Fe2	175.35 (17)		
			
C5—C1—C2—C3	0.6 (2)	C1—C5—Fe1—O3	−118.19 (11)
Fe1—C1—C2—C3	−60.70 (13)	C4—C5—Fe1—C2	−81.72 (12)
C5—C1—C2—Fe1	61.25 (13)	C1—C5—Fe1—C2	38.52 (11)
C1—C2—C3—C4	−0.4 (2)	C4—C5—Fe1—C3	−37.96 (11)
Fe1—C2—C3—C4	−61.56 (13)	C1—C5—Fe1—C3	82.28 (12)
C1—C2—C3—Fe1	61.13 (13)	C4—C5—Fe1—C1	−120.24 (16)
C2—C3—C4—C5	0.2 (2)	C1—C5—Fe1—C4	120.24 (16)
Fe1—C3—C4—C5	−60.48 (13)	C11—C10—Fe2—C14	−145.14 (13)
C2—C3—C4—Fe1	60.63 (13)	C9—C10—Fe2—C14	96.58 (13)
C3—C4—C5—C1	0.2 (2)	C11—C10—Fe2—C15	−47.42 (14)
Fe1—C4—C5—C1	−58.91 (13)	C9—C10—Fe2—C15	−165.69 (12)
C3—C4—C5—Fe1	59.11 (13)	C11—C10—Fe2—O4	112.87 (15)
C2—C1—C5—C4	−0.5 (2)	C9—C10—Fe2—O4	−5.4 (2)
Fe1—C1—C5—C4	59.26 (13)	C9—C10—Fe2—C11	−118.28 (17)
C2—C1—C5—Fe1	−59.72 (12)	C11—C10—Fe2—C9	118.28 (17)
C13—C9—C10—C11	0.8 (2)	C11—C10—Fe2—C12	37.90 (12)
Fe2—C9—C10—C11	−60.69 (14)	C9—C10—Fe2—C12	−80.38 (13)
C13—C9—C10—Fe2	61.45 (13)	C11—C10—Fe2—C13	80.73 (13)
C9—C10—C11—C12	0.2 (2)	C9—C10—Fe2—C13	−37.55 (12)
Fe2—C10—C11—C12	−60.52 (14)	C10—C11—Fe2—C14	41.31 (15)
C9—C10—C11—Fe2	60.72 (13)	C12—C11—Fe2—C14	160.03 (12)
C10—C11—C12—C13	−1.1 (2)	C10—C11—Fe2—C15	140.10 (12)
Fe2—C11—C12—C13	−61.14 (13)	C12—C11—Fe2—C15	−101.18 (13)
C10—C11—C12—Fe2	60.05 (14)	C10—C11—Fe2—O4	−130.14 (13)
C10—C9—C13—C12	−1.5 (2)	C12—C11—Fe2—O4	−11.43 (18)
Fe2—C9—C13—C12	58.58 (14)	C12—C11—Fe2—C10	118.72 (17)
C10—C9—C13—Fe2	−60.04 (13)	C10—C11—Fe2—C9	−38.31 (12)
C11—C12—C13—C9	1.6 (2)	C12—C11—Fe2—C9	80.41 (13)
Fe2—C12—C13—C9	−58.41 (14)	C10—C11—Fe2—C12	−118.72 (17)
C11—C12—C13—Fe2	59.99 (13)	C10—C11—Fe2—C13	−81.09 (13)
C3—C2—Fe1—C6	−46.86 (14)	C12—C11—Fe2—C13	37.62 (12)
C1—C2—Fe1—C6	−165.13 (12)	C13—C9—Fe2—C14	158.36 (13)
C3—C2—Fe1—C7	−139.46 (12)	C10—C9—Fe2—C14	−83.87 (13)
C1—C2—Fe1—C7	102.27 (12)	C13—C9—Fe2—C15	−84.2 (2)
C3—C2—Fe1—O3	123.20 (13)	C10—C9—Fe2—C15	33.6 (3)
C1—C2—Fe1—O3	4.93 (17)	C13—C9—Fe2—O4	59.31 (13)
C1—C2—Fe1—C3	−118.27 (16)	C10—C9—Fe2—O4	177.08 (11)
C3—C2—Fe1—C1	118.27 (16)	C13—C9—Fe2—C10	−117.77 (17)
C3—C2—Fe1—C4	38.15 (11)	C13—C9—Fe2—C11	−80.10 (13)
C1—C2—Fe1—C4	−80.12 (12)	C10—C9—Fe2—C11	37.67 (12)
C3—C2—Fe1—C5	80.75 (12)	C13—C9—Fe2—C12	−36.25 (12)
C1—C2—Fe1—C5	−37.52 (11)	C10—C9—Fe2—C12	81.52 (13)
C2—C3—Fe1—C6	141.09 (12)	C10—C9—Fe2—C13	117.77 (17)
C4—C3—Fe1—C6	−101.01 (12)	C13—C12—Fe2—C14	73.1 (2)
C2—C3—Fe1—C7	46.98 (14)	C11—C12—Fe2—C14	−44.7 (3)
C4—C3—Fe1—C7	164.89 (11)	C13—C12—Fe2—C15	−160.72 (12)
C2—C3—Fe1—O3	−119.34 (13)	C11—C12—Fe2—C15	81.44 (13)
C4—C3—Fe1—O3	−1.44 (18)	C13—C12—Fe2—O4	−69.34 (12)
C4—C3—Fe1—C2	117.90 (16)	C11—C12—Fe2—O4	172.83 (12)
C2—C3—Fe1—C1	−38.12 (11)	C13—C12—Fe2—C10	80.34 (13)
C4—C3—Fe1—C1	79.78 (12)	C11—C12—Fe2—C10	−37.50 (12)
C2—C3—Fe1—C4	−117.90 (16)	C13—C12—Fe2—C11	117.83 (18)
C2—C3—Fe1—C5	−80.90 (12)	C13—C12—Fe2—C9	36.20 (12)
C4—C3—Fe1—C5	37.01 (11)	C11—C12—Fe2—C9	−81.63 (13)
C5—C1—Fe1—C6	−82.2 (2)	C11—C12—Fe2—C13	−117.83 (18)
C2—C1—Fe1—C6	35.7 (3)	C9—C13—Fe2—C14	−28.18 (16)
C5—C1—Fe1—C7	162.86 (12)	C12—C13—Fe2—C14	−149.00 (13)
C2—C1—Fe1—C7	−79.23 (12)	C9—C13—Fe2—C15	147.70 (13)
C5—C1—Fe1—O3	65.06 (12)	C12—C13—Fe2—C15	26.88 (17)
C2—C1—Fe1—O3	−177.03 (11)	C9—C13—Fe2—O4	−125.46 (12)
C5—C1—Fe1—C2	−117.91 (16)	C12—C13—Fe2—O4	113.72 (12)
C5—C1—Fe1—C3	−80.07 (12)	C9—C13—Fe2—C10	38.75 (12)
C2—C1—Fe1—C3	37.84 (11)	C12—C13—Fe2—C10	−82.07 (13)
C5—C1—Fe1—C4	−36.39 (11)	C9—C13—Fe2—C11	82.29 (13)
C2—C1—Fe1—C4	81.52 (12)	C12—C13—Fe2—C11	−38.53 (12)
C2—C1—Fe1—C5	117.91 (16)	C12—C13—Fe2—C9	−120.82 (17)
C5—C4—Fe1—C6	−159.28 (12)	C9—C13—Fe2—C12	120.82 (17)
C3—C4—Fe1—C6	81.99 (12)	O4—C8—O3—Fe1	2.3 (2)
C5—C4—Fe1—C7	86.2 (2)	C6—Fe1—O3—C8	−47.85 (15)
C3—C4—Fe1—C7	−32.6 (2)	C7—Fe1—O3—C8	45.98 (15)
C5—C4—Fe1—O3	−62.12 (12)	C2—Fe1—O3—C8	140.73 (15)
C3—C4—Fe1—O3	179.16 (10)	C3—Fe1—O3—C8	−146.20 (15)
C5—C4—Fe1—C2	80.61 (12)	C1—Fe1—O3—C8	144.01 (14)
C3—C4—Fe1—C2	−38.11 (11)	C4—Fe1—O3—C8	−147.16 (14)
C5—C4—Fe1—C3	118.72 (16)	C5—Fe1—O3—C8	179.01 (15)
C5—C4—Fe1—C1	36.58 (11)	O3—C8—O4—Fe2	−174.87 (13)
C3—C4—Fe1—C1	−82.14 (12)	C14—Fe2—O4—C8	−40.67 (17)
C3—C4—Fe1—C5	−118.72 (16)	C15—Fe2—O4—C8	−138.29 (16)
C4—C5—Fe1—C6	28.79 (16)	C10—Fe2—O4—C8	58.8 (2)
C1—C5—Fe1—C6	149.03 (12)	C11—Fe2—O4—C8	131.88 (16)
C4—C5—Fe1—C7	−143.93 (12)	C9—Fe2—O4—C8	55.10 (17)
C1—C5—Fe1—C7	−23.69 (16)	C12—Fe2—O4—C8	124.36 (16)
C4—C5—Fe1—O3	121.58 (11)	C13—Fe2—O4—C8	88.04 (16)

## References

[bb1] Bruker (2007). *APEX2*, *SADABS*, *SAINT-Plus* and *XPREP* Bruker AXS Inc., Madison, Wisconsin, USA.

[bb2] Darensbourg, D. J., Day, C. S. & Fischer, M. B. (1981). *Inorg. Chem.* **20**, 3511–3519.

[bb3] Darensbourg, D. J., Fischer, M. B., Raymond, J., Schmidt, E. & Baldwin, B. J. (1981). *J. Am. Chem. Soc.* **103**, 1297–1298.

[bb4] Dombek, B. D. & Angelici, R. J. (1973). *Inorg. Chem.* **7**, 345–347.

[bb5] Farrugia, L. J. (1997). *J. Appl. Cryst.* **30**, 565.

[bb6] Farrugia, L. J. (1999). *J. Appl. Cryst.* **32**, 837–838.

[bb7] M’thiruaine, C. M., Friedrich, H. B., Changamu, E. O. & Bala, M. D. (2011). *Inorg. Chim. Acta*, **366**, 105–115.

[bb8] M’thiruaine, C. M., Friedrich, H. B., Changamu, E. O. & Omondi, B. (2011). *Acta Cryst.* E**67**, m485. 10.1107/S1600536811010154 PMC3099767 21753996

[bb9] Pinkes, J. R., Masi, C. J., Chiulli, R., Steffey, B. D. & Cutler, A. R. (1997). *Inorg. Chem.* **36**, 70–79.

[bb10] Sheldrick, G. M. (2008). *Acta Cryst.* A**64**, 112–122. 10.1107/S0108767307043930 18156677

[bb11] Tso, C. C. & Cutler, A. R. (1985). *Organometallics*, **4**, 1242–1247.

[bb12] Tso, C. C. & Cutler, A. R. (1990). *Inorg. Chem.* **29**, 471–475.

